# High-throughput novel microsatellite marker of faba bean via next generation sequencing

**DOI:** 10.1186/1471-2164-13-602

**Published:** 2012-11-08

**Authors:** Tao Yang, Shi-ying Bao, Rebecca Ford, Teng-jiao Jia, Jian-ping Guan, Yu-hua He, Xue-lian Sun, Jun-ye Jiang, Jun-jie Hao, Xiao-yan Zhang, Xu-xiao Zong

**Affiliations:** 1 Institute of Crop Science, The National Key Facility for Crop Gene Resources and Genetic Improvement, Chinese Academy of Agricultural Sciences, Beijing, 100081, China; 2 Institute of Grain Crops, Yunnan Academy of Agricultural Sciences, Kunming, 650205, China; 3 Department of Agriculture and Food Systems, Melbourne School of Land and Environment, The University of Melbourne, Melbourne, Victoria, 3010, Australia; 4 Qingdao Academy of Agricultural Sciences, Qingdao, 266100, China

**Keywords:** Microsatellite markers, Next generation sequencing, Marker development, *Vicia faba* L.

## Abstract

**Background:**

Faba bean (*Vicia faba* L.) is an important food legume crop, grown for human consumption globally including in China, Turkey, Egypt and Ethiopia. Although genetic gain has been made through conventional selection and breeding efforts, this could be substantially improved through the application of molecular methods. For this, a set of reliable molecular markers representative of the entire genome is required.

**Results:**

A library with 125,559 putative SSR sequences was constructed and characterized for repeat type and length from a mixed genome of 247 spring and winter sown faba bean genotypes using 454 sequencing. A suit of 28,503 primer pair sequences were designed and 150 were randomly selected for validation. Of these, 94 produced reproducible amplicons that were polymorphic among 32 faba bean genotypes selected from diverse geographical locations. The number of alleles per locus ranged from 2 to 8, the expected heterozygocities ranged from 0.0000 to 1.0000, and the observed heterozygosities ranged from 0.0908 to 0.8410. The validation by UPGMA cluster analysis of 32 genotypes based on Nei's genetic distance, showed high quality and effectiveness of those novel SSR markers developed via next generation sequencing technology.

**Conclusions:**

Large scale SSR marker development was successfully achieved using next generation sequencing of the *V. faba* genome. These novel markers are valuable for constructing genetic linkage maps, future QTL mapping, and marker-assisted trait selection in faba bean breeding efforts.

## Background

Faba bean (*Vicia faba* L.) is an important temperate legume, grown for human consumption and animal feed due to its high protein and fibre content [1,2]. The crop also replaces available nitrogen in the soil when used in rotation with cereals and oilseeds, and thus is expected to be a highly beneficial component in future temperate Low Carbon Agricultural systems. China is the largest faba bean producer (40.36%) with an average dry grain production (2005–2009) of 1,720,000 metric tonnes (mt) from 945,400 hectares; followed by Ethiopia (476,026 mt), France (331,122 mt), Egypt (274,040 mt) and Australia (196,800 mt) [3].

However, faba bean suffers from several major biotic and abiotic factors that constrain productivity. Although significant genetic gain to overcome these has been made through traditional breeding practices [1], progress through the use of genomics and associated biotechnologies is limited. This is due mainly to the large genome size (13GB; [4]), which is approximately 25 times larger than that of the model legume *Medicago truncatula*, and 2.5 times larger than *Pisum sativum*[1], together with a lack of financial investment in this crop species.

Recent advances in next generation sequencing (NGS) technologies enable the generation of large volumes of sequence efficiently and cost-effectively [5,6]. This has led to a revolution in biological and agricultural applications including identification of genes correlated with key breeding traits through high-density SNP marker and genome-wide association analysis studies (GWAS) [7,8]. Another outcome is the ability to accurately identify sequences flanking simple sequence repeat (SSR) regions for use as locus-specific markers for downstream genotyping. Otherwise known as microsatellites, SSRs are tandemly repeated motifs of 1 to 6 nucleotides found in both coding and non-coding regions [9,10]. These have become a marker of choice in many genotyping applications due to their relatively high abundance, high level of allelic variation, co-dominant inheritance, analytical simplicity and transferability of results across laboratories [11].

A limited number of characterized SSR loci (<120) which have been validated over relatively few genetic backgrounds are available for faba bean. Initially, Pozarkova *et al*. developed primers to 25 SSR loci detected in chromosome 1 DNA libraries [12]. Subsequently, Zeid *et al*. developed primers to 54 SSR loci [13] and Gong *et al*. developed 11 EST-SSR loci primers [14]. Most recently, EST sequences within the public domain databases were screened and an additional 21 novel SSR loci were characterized and validated among 32 faba bean accessions [15].

Besides providing a cost-effective valuable source for molecular marker generation, the identification of SSR within ESTs is an effective approach for gene discovery and transcript pattern characterization, particularly if through mapping an EST-SSR or EST marker is significantly associated with a QTL [16-18]. This may be achieved by searching for SSR associated sequences within EST of a well characterised crop or model plant species. Together with the advantage of *in silico* analysis, this approach has the potential to substantially broaden the field of comparative studies to species where limited or no sequence information is available.

The present study identified high-quality putative SSR loci and flanking primer sequences cheaply and efficiently using the Roche 454 GS FLX Titanium platform. The resultant SSR sequences were characterized and validated through successful amplification of randomly selected target loci across a selection of faba bean genotypes from diverse geographic origin.

## Methods

### Plant material

A total of 247 faba bean accessions were selected from the National Genebank of China held at the Institute of Crop Science (ICS), Chinese Academy of Agricultural Sciences (CAAS), Beijing. Of these, 100 originated from China, 54 were from other Asian countries, 39 were from Europe, 30 were from Africa, 14 were from the America, 9 breeding lines were sourced from the ICARDA (International Center for Agricultural Research in the Dry Areas) faba bean breeding program and one was from Oceania (Additional file 1: Table S1).

### DNA isolation, library preparation and 454 sequencing

Seven days after seed were left on moist filter paper in the dark at 22°C, sprouts from each of the 247 genotypes were collected. A single sprout of each genotype and of approximately the same weight was pooled and total gDNA was extracted using the CTAB method [19,20].

Genome libraries were constructed using eight biotin labeled probes and a selective hybridization with streptavidin coated bead method [21-23]. The probes were: pGA, pAC, pAAT, pAAC, pAAG, pATGT, pGATA and pAAAT. The quality of libraries was inspected by randomly selecting and sequencing 276 clones. The cloning vector was pEASY-T1 (TransGen Biotechnology Co., Ltd), and the primers used for sequencing were F: 5^′^-GTAAAACGACGGCCAGT-3^′^ and R: 5^′^-CAGGAAACAGCTATGAC-3^′^. Libraries were considered to be of high quality if the length of sequences were from 200 to 1000 bp, as evidenced on agarose gel.

Subsequently, entire libraries were equally pooled and subjected to 454 sequencing with GS-FLX Titanium reagents at Beijing Autolab Biotechnology Co., Ltd (China). All processing and analyses of the sequencing data was performed with GS-FLX Software v2.0.01 (454 Life Sciences, Roche, Germany). Using a series of normalization, correction and quality-filtering algorithms, the 454 sequencing data were processed to screen and filter for weak signals and low-quality reads, and to trim the read ends for 454 adaptor sequences using the EMBOSS [24] software package. The sequencing data were then submitted to the ^′^National Center for Biotechnology Information (NCBI) short read archive and given the accession number SRP006387.

### SSR loci search and primer design

The software MISA (Microsatellite identification) tool (http://pgrc.ipk-gatersleben.de/misa/) was configured to locate a minimum of 10 bp: monomers (×10), 2-mers (×6), 3-mers (×5), 4-mers (×5), 5-mers (×5) and 6-mers (×5). This tool allowed the identification and localization of perfect microsatellites as well as compound microsatellites. The maximum size of interruption allowed between two different SSR in a compound sequence was 100 bp. Subsequently, Primer 3.0 (http://www-genome.wi.mit.edu/genome_software/other/primer3.html.) was used to design primer pairs to the flanking sequences of each unique SSR.

### SSR characterization and validation

The number of different types of SSR, length (motif bp × number of motifs) and SSR position was searched and analyzed for using a bespoke program written in MISA files [25] and plotted by OpenOffice.org Calc.

### Marker assessment

Polymerase chain reactions (PCR) were performed in 20 μl reaction volumes containing 0.5 U of *Taq* DNA polymerase (Zhexing, Beijing, China), 1 × PCR BufferII, 1.5 mM MgCl_2_, 25 μM of dNTP, 0.4 μM primer, and 50 ng of genomic DNA. Microsatellite loci were amplified on a Heijingang Thermal Cycler (Eastwin, Beijing, China) with the following cycle: 5 min initial denaturation at 95°C; 35 cycles of 30s at 95°C, 30s at the optimized annealing temperature (Table 1), 45s of elongation at 72°C, and a final extension at 72°C for 10min. PCR products were initially assessed for size polymorphism on 6% denaturing polyacrylamide gels and visualized by silver nitrate staining.

**Table 1:** Occurrence of microsatellites in the genome survey

**Category**	**Numbers**
Total number of sequences examined	532,599
Total size of examined sequences (bp)	162,448,842
Total number of identified SSRs	250,393
Number of SSR containing sequences	125,559
Number of sequences containing more than one SSR	61,266
Number of SSRs present in compound formation	122,988

The genotyping data was subsequently used to determine genetic relationships among 32 *V. faba* accessions (eleven from China, seven from Asia, five from Europe, five from Africa, three from the Americas and one from Oceania; (Additional file 1: Table S1). The number of alleles (*Na*), expected (*He*) heterozygosities and observed (*Ho*) heterozygosities were calculated using POPGEN1.32 [26]. The cluster analysis of 32 genotypes was carried out based on Nei's unbiased measures of genetic distance [27] by using the unweighted pair-group method with arithmetic average (UPGMA), and the dendrogram was drawn by MEGA4 [28].

## Results

### Quality inspection of the DNA library

The recombination rate within the constructed SSR-enriched *V. faba* library was 73.9%. Among the 276 clones sequenced, 31.9% contained SSR sequences within an insert that ranged from 0.2 to 1.0 kb in size.

### 454 sequencing and characterization reads

**Figure 1:**
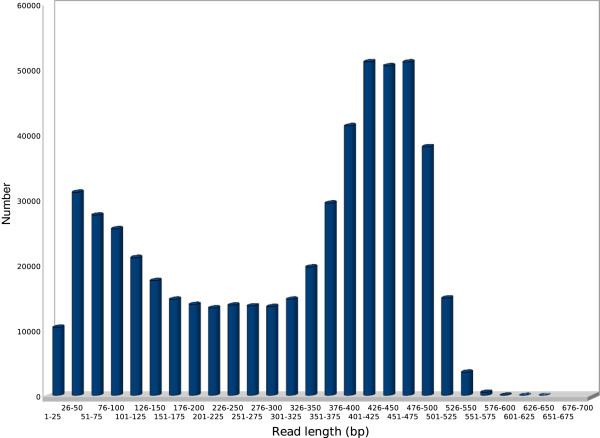
Frequencies length distribution of 454 read sequences.

A total of 578,251 reads were generated from the pooled library, and 532,599 read sequences were used for further analysis after adaptor removal. Adenine was the most abundant nucleotide (30%), followed by thymine (27%), guanine (22%) and cytosine (21%). The mean GC content was 43%. The average length of read sequence was 305 bp, with a maximum length of 635 bp (Figure 1).

### Identification of SSR loci

After MISA analysis, the number of sequences containing an SSR was 125,559, and in total 250,393 SSR loci were detected. The number of sequences containing more than one SSR loci was 61,266 and the number of SSRs present in compound formation was 122,988 (Table 1).

**Figure 2:**
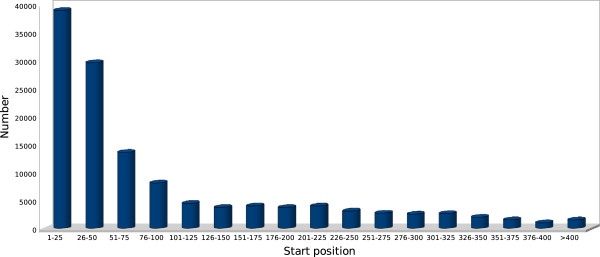
**The frequency of the SSR motif start position from the 5’ terminus of the cloned insert within the enriched libraries** .

The total size of SSR motif sequences was 8,759,185 bp, with an average motif length of 69 bp. Of these, 25% comprised more than one discrete repeat and a high proportion (49%) was located within compound repeats. The majority of identified SSR motifs (83%) were located between the 5’-terminus and mid regions of the cloned sequences, and within 200 bp of the 5’-terminus (Figure 2). A total of 28,503 primer pairs were designed for future assessment of locus amplification (Additional file 2: Table S2).

### Abundance and length frequencies of SSR repeat motifs

**Figure 3:**
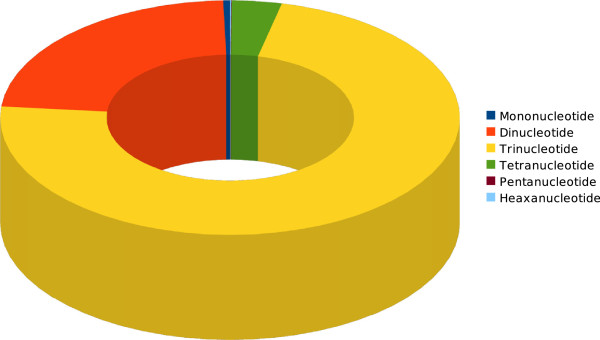
**Frequencies of different nucleotide repeat sizes within the clones analysed** .

The most common SSR motifs comprised trinucleotide and dinucleotide repeats (Figure 3). The majority of the trinucleotide repeats were from 15 to 30 bp in length. Within the 1,188 characterised mononucleotide SSR, (A/T)_n_ was almost three times more common than (C/G)_n_, particularly at the 11–12 bp length. The dinucleotide repeats (AC/GT)_n_ and (AG/CT)_n_ were predominant, representing 99.2% of all of the dinucleotides characterised. Triucleotide (AAC/GTT)_n_ repeats were the most abundant (96.5%). Twenty two unique tetranucleotide repeat motifs were identified, with the most common being AGAT/ATCT (66.4%), ACAG/CTGT (19.3%) and ACAT/ATGT (9.1%). Pentanucleotide and hexanucleotide motifs were far less frequent, together comprising only 0.1% of the total SSR detected. The dominant pentanucelotide motif was AGAGT/ATCTC (23.8%) and the most common hexanucelotide motif was ACACGC/CGTGTG (49.5%) (Additional files 3, 4, 5, 6, 7 and 8: Figure S1-S6).

### Compound SSR analysis

Two types of compound SSR were identified; those without an interruption between two motifs (ie (CA)12(ACG)37 and noted as C* type) and those with an interruption between two motifs ( ie (AAC)7gtcaat(AAC)5 and noted as C type). In total, 1,893 C* type and 59,369 C type compound SSR loci were detected among those sequenced, reflecting the complexity of the faba bean genome.

### Validation of SSR assay

Of the 150 primer pairs selected for validation of SSR locus amplification, 102 produced a reproducible and clear amplicon of the expected size. Of these, 94 (63%) were polymorphic among thirty-two genotypes assessed (Table 2). The number of alleles per locus ranged from 2 to 8, the expected heterozygosities ranged from 0.0000 to 1.0000, and the observed heterozygosities ranged from 0.0908 to 0.8410 (Table 3).

**Table 2:** **Characteristics of 94 polymorphic SSR markers developed in *Vicia faba *L. (F=forward primer, R=reverse primer, Size = size of cloned allele, Ta = annealing temperature)**

**Primer**	**Repeat**	**F (5’– 3’)**	**R (5’– 3’)**	**Size (bp)**	***Ta*(°C)**
CAAS1	(AAAGGG)7	AGTCAGGGGGTCGATTTTTC	TCTTGCGCAGTTTTGACATC	212	55
CAAS2	(GAA)9	TACAAAAGCTCTGGGGCCTA	CCAATTCCTCTGGGCAACT	202	56
CAAS3	(AG)7	CTGGTGCGTAAGGTTGATGA	CAAACCACCACCAATCACAG	132	53
CAAS4	(CA)11	ATTGCAAGTCCTGAGGCAAG	ATAATGGCGCCACAAAGTGT	160	57
CAAS5	(ACA)15	TACATCAGTCCCGCAAATCA	CCATGTAGCCGATTCCACTT	150	55
CAAS6	(A)10	TGCAAAGTAATTCCGAAACAA	CGCACATGAATTGGGGTAAT	150	56
CAAS7	(A)10	GACCCAAGCCTTCACCACTA	TGTGTGGGATCCATTTTGAA	200	59
CAAS8	(AAC)14	AATTTGTTCAGCATCTCGGG	CTGGTTGGTTCCTGGTGAGT	150	56
CAAS9	(AAC)9	GTGATGCTTTGCCTGTGCTA	ATGGACGTTTGTAGGTGGGA	200	56
CAAS10	(AAG)5	CTGTTCGTCATCATCATCGG	CGTAAATCAACCCCAACACC	150	53
CAAS11	(ACA)10	TCCCGCTATTCTTGCTCTGT	GCTCAAAAATGCTTGTCTTTCA	170	54
CAAS12	(TGT)9	GAGGAGGATCCCACAATGAA	GCCAAAAGAGCCATGGTAGA	210	56
CAAS13	(CAA)11aaatcccaaaaactgcaaattgtatgccatcttaaaccatac(CAA)7	CAAAAATCCCAAAAACTGCAA	TCGATTTTTCGACTTGGGTC	130	56
CAAS14	(AAC)6	CCGTAGATCTCAAAAACCATGA	GGAGGAAGGAAGCTCGAATC	170	60
CAAS15	(AAC)8	AACCAACATCAATGGCATCA	TCTTTTCCTTTTTCCTCTTCCA	140	60
CAAS16	(CA)7	TCAAATTTCCCTTTGCAAAAAT	GACCAAGGTCAACCACCTTT	350	56
CAAS17	(CA)8	TCAAACACCTACACACCCACA	TCTCGGTCAATCTCACATGC	250	56
CAAS18	(CA)9	ATGGGAGGGCAAATTTTAGG	AGTGAGTGGAGCGCTTGTTT	350	56
CAAS19	(CAA)6	AACATTTTTCCAATCGAGGC	TGTAGGCTTACGGCCAAAGA	200	56
CAAS20	(CAT)5	ACTGGAAAATCCCAATGCAC	AGCAAACTTGCACCCAACAT	190	56
CAAS21	(CTT)8	GAATTTTCAAAACATGAGTCCCA	CCGGATCTGAAAAGACTTGC	175	60
CAAS22	(G)10	TGATGAACAGAACTGCGCTC	ATTGGAGAGAGGCGAAATCA	190	56
CAAS23	(GA)6	ACCGCATGCTAGGGAGTCTA	TGGGTGACTCACTTTTGTGG	220	58
CAAS24	(GA)6gca(AG)6(TG)8	TCACTCACAAGCCACTAAGTCAA	GATGCGACACTATCCCCACT	200	56
CAAS25	(GT)15	TCCATAATCAATTGGCTAAGCTC	AAGACTAACTCTCGACTGTATTTAGGC	150	58
CAAS26	(GT)7	CGGCTTGGTTAACTGGATGT	TCTTCCTTTTCTTCAATGCG	160	58
CAAS27	(TA)6	TTGGCATCATGCTCTAATCG	CTTGAAGTCGTGCCAGATGA	280	60
CAAS28	(TC)8	CCATTGATGCAGGAAAGGAT	CAGCTTTGACAGCTCCAACA	160	58
CAAS29	(TCA)5	TGCAAGTCAGTAGCCAAGACA	CTCGTCTCTCCTCATTCCCA	180	58
CAAS30	(TG)10	GGTTTTTAGGTGATTTTCGCA	GCGAAACCTCGTATGGTTGT	170	59
CAAS31	(TG)12	CAACGCGCTAGAGGAAGAAG	CCACTGCCCTAGCACACTAA	160	56
CAAS32	(TG)7	TTTGGGGTACAACACTGGGT	CCTCACTCCTCTATATAAACAACACTT	200	59
CAAS33	(TGA)5	GCAGTGATTCTGGCAGTGAA	TGCAGCAACATTTCCATCAT	190	56
CAAS34	(TGT)5	TTTCTCGCAATTGTTCTCACA	TTCGATGAAATCCATCTTCTGA	200	57
CAAS35	(TTG)8	AGGCAGAAGTTTGGAAGCAA	TCTCACTTCGGCTTCAGGAT	180	56
CAAS36	(A)11	AGCACTAGAGTTCCAAGCCA	TTTTTATCGTTTCTTGTCACGC	130	52
CAAS37	(A)11	CAACGCAAGAACACGTGAAT	TAGAGGCCAATTCAAGCCAT	190	54
CAAS38	(AAC)5	CGCCTCAGAACCAAGTTCAT	TGCTTTGTTTTGGTTTTGTGA	170	56
CAAS39	(AAG)5	CTGTTCGTCATCATCATCGG	CGTAAATCAACCCCAACACC	170	54
CAAS40	(AAG)6	CCAAAGCCACTTCCAAACAT	TTCAGCCGGGCTTCTTTC	110	54
CAAS41	(AC)10	GAAACCCACTTGGTCGTGTC	TTCATTTGGGTAGGCTCCAA	190	56
CAAS42	(AC)10	CAAGTGTCGACGCAAGAGAT	TGACTTTTTGACTGCTCCCA	250	56
CAAS43	(AC)7	GAGGAAGTGTGAAAGGTCGC	TCATTTTAAAGTGGTGTATGTGTGT	170	54
CAAS44	(AC)7	ACACACACACGCACACACAC	CATGAACCTTTGATAGTTTTCCA	150	56
CAAS45	(AGA)5	ATGGCTTTGACAAAAGGGAA	CTCCTTCACCCGACAATGTT	180	57
CAAS46	(AGA)6	AGATCGCAGGCGTAGAAAGA	TGCTTCAACCACAACACCAT	200	58
CAAS47	(C)11	CAAATTGGTTTGCATATCCG	AGCCCTTCACATCCATTGAG	200	56
CAAS48	(CA)10	CCTCCTCCTTTAATTTGTGGC	TGAATCGTGAATGCTCTCTGA	200	56
CAAS49	(CA)10	ACCTCCATAGCAGCAGCATT	GGCCAATTCTTAACGTGCTT	140	56
CAAS50	(CA)10	CACTGGACCATTTTGCATTC	ATGAGATCCGGAGCAGATGT	140	56
CAAS51	(CA)11	AAGCATTAAAACTCCCATAGCG	ATGTGTGCGTGTGTCATGTG	140	52
CAAS52	(CA)12	CATTCCATGTTGCGTTTTTG	GGATAAGAGGGTGGTGGTGA	200	56
CAAS53	(CA)13	GGCCCATTTGTTAAGGGTTT	AATGAGATCTGGCCTGGATG	200	56
CAAS54	(CA)6	CCATTGGACCTCTTTGCATT	CCAGAGTGGATGATGATCTGA	150	54
CAAS55	(CA)6	ACTCACATACACGCACACACA	AATGCTCTCATCCCTTTTGC	150	56
CAAS56	(CA)6	CACATACACGCACACACACA	AATGCTCTCATCCCTTTTGC	150	56
CAAS57	(CA)8	GCCCGAGACACTTTGGTTTA	CCAGAATGGATGAGGACCTG	210	56
CAAS58	(CA)9	CTCCTGGTCCATGTATGAATGA	TGTGTGTATGTGTATGCGTGC	150	54
CAAS59	(CAA)10	GGCCAACATAGGTGAGCATT	GTGTTGTAGGCCTTTGGTCC	200	56
CAAS60	(CAA)8	ATGCAAAATGAAATGCGACA	TGTAGTTGTCTGTTTAATGGTTGTTG	190	56
CAAS61	(G)11	AGAGGAAAAAGGCAAATGGC	CCCTTCATCAATCACACCAA	130	54
CAAS62	(GA)14	AATGTTGGGACGGAGTTCAG	TTGTTGATTCATTCATCCCTTG	130	56
CAAS63	(GA)15	CGCAGAGAAACACTCCATGA	GAAGTTGAATGTCATTTGTGTCAA	100	56
CAAS64	(GA)6	AAAATATAATAAACAAAGCAAAAGTGC	CAGGTTTGTGGTTTCACCCT	200	54
CAAS65	(GA)6	CGATATTCCTCGGTTTCCAA	CATGGGTCGTCTTCTCCACT	200	54
CAAS66	(GA)6	CATCACTTTCCAGCCTGTCA	ATTTTCTGCCTCCCCTTTGT	190	58
CAAS67	(GA)7	GGGTTTCAGAGAAAGGGGTC	CGCAAGCGTATTGGGTATTT	130	56
CAAS68	(GA)8	ATGGAGGTTGCGATTTGAAG	CATCATCTCCACACTTTTTCCA	130	54
CAAS69	(GT)10	ATTACAAATGTCGGTGCCGT	AGCACAACGATAAGATGATATGC	170	54
CAAS70	(GT)8	TCGCGATAGAGGTTTTGGAA	AACAACAACGATTCATCACAAGA	200	56
CAAS71	(GTT)15	CCATGTAGCCGATTCCACTT	TTCGGCAACGTAGGAAAAAT	160	54
CAAS72	(T)10	TTTTCCAGTGTCAACCCATCT	ACATGAGGCCAAAAACTGCT	170	54
CAAS73	(TG)13	TTGCACCTCTGTTGAAGACG	TCACCAACACTCTAATCCTCAATC	190	54
CAAS74	(AC)6	CCCACCGTATTACACAAGGG	GCGAGGAAGAAGATGACGTT	200	56
CAAS75	(AG)15	TCGATTGCACAATAAATGGTTT	GAGGTCGACTCCCATTGAAA	180	54
CAAS76	(AG)6	GCCTGTTAATGAGAAGAACTGGA	TTTCAAAATTTAGTTTCTCTCTGTCTC	200	56
CAAS77	(CA)21	TAGCAGCCAACAATCAGTGG	GGTGATGTTGCTCATGTTCG	180	56
CAAS78	(CA)7	TCAAATTTCCCTTTGCAAAAAT	TCGAACACAACTTCTTCATTTCTC	180	56
CAAS79	(CA)7	TCAAATTTCCCTTTGCAAAAAT	CATGGAAAATCTTTTATTTTGTGTG	100	58
CAAS80	(CA)8	GTGTGAAAACTCACCCGGTC	TGTGTGTAAGTGTGTGTATGTGTGTG	130	54
CAAS81	(GA)15	AACTTACAGGGGCCACACAC	TGTGCATTATACTTTACGTATGTTCCT	100	52
CAAS82	(GA)17	TTTGCTTGACAATGGTGGAA	ATTCAACAAGCAAGGGTTGG	120	52
CAAS83	(T)10	GATTTGCGTTTAGGGTTCCA	GAACAAACTACGTTTTATTGTCCAGA	180	52
CAAS84	(TA)6	TGTCGACACCACAGCTATTTT	TGTGGTTCGTTGTTTTGGTG	200	56
CAAS85	(TCA)6	TTGAAGTGAATAAGATGAAGAAGTGT	GTTGCCTTTCCTTGCATGAT	130	56
CAAS86	(TG)10	TCGCGATAGAGGTTTTGGAA	CACAAACAACAACGATTCATCA	200	56
CAAS87	(TG)14	CTCTACCATGGGCCATTTCT	AGAGATAGAGAGAGAGACAGAGATGAA	90	54
CAAS88	(TG)18	TCCTACCGATCTCTCTCTCCC	GTGGCATAACCGCGTAAGTT	130	56
CAAS89	(TG)18	TGTCTCGCCTTCAATCTTCC	CTTGCTAAGTGAGACTGCTGCT	190	54
CAAS90	(TG)19	TCCATAGTCGATGAGGACCG	TTGTCTCATTGTCTTTCTTTTCTTTC	100	54
CAAS91	(TG)6	ATCTTCGGCTTGGTTGATTG	GAGGCGGCCACATTAGACT	200	56
CAAS92	(TG)9	CGAGATCTGGAGTGGATTTAGA	TTTTCATATGCCACATGCTCA	170	56
CAAS93	(TTC)5	GGCATTGCTTACTTACCGGA	CGACGTCGACATTAACATGC	200	56
CAAS94	(TTG)9	TCCTCAACACGTGATGCAAT	TGTAGGACCAGGAAGGTCGT	180	56

**Table 3:** **Informativeness of SSR loci following amplification from 32 geographically diverse accessions of *Vicia faba *L**

**Locus**	**32 Accessions**		
	***Na***	***He***	***Ho***
CAAS1	3	0.0000	0.3591
CAAS2	3	0.2857	0.5703
CAAS3	7	0.4444	0.8099
CAAS4	4	0.0000	0.6111
CAAS5	3	0.1111	0.6471
CAAS6	4	0.2188	0.6324
CAAS7	6	0.6774	0.7372
CAAS8	7	0.6250	0.8016
CAAS9	4	0.1290	0.7250
CAAS10	4	0.7419	0.7277
CAAS11	4	0.3929	0.6890
CAAS12	4	0.1000	0.6718
CAAS13	5	0.3871	0.6256
CAAS14	3	0.4062	0.6493
CAAS15	4	0.6129	0.6901
CAAS16	6	0.6667	0.7708
CAAS17	3	0.0000	0.5159
CAAS18	4	0.3333	0.6887
CAAS19	5	0.0500	0.7474
CAAS20	4	0.2593	0.5926
CAAS21	4	0.1562	0.4712
CAAS22	3	0.2222	0.6038
CAAS23	2	0.0938	0.0908
CAAS24	6	0.1000	0.8000
CAAS25	5	0.4375	0.7399
CAAS26	3	0.0000	0.6333
CAAS27	5	0.2963	0.7701
CAAS28	4	0.5294	0.6471
CAAS29	4	0.3793	0.4483
CAAS30	4	0.2917	0.4991
CAAS31	4	0.4167	0.3608
CAAS32	5	0.6875	0.7882
CAAS33	3	0.2188	0.6195
CAAS34	3	0.4091	0.5613
CAAS35	4	0.3226	0.6753
CAAS36	3	0.3182	0.6131
CAAS37	2	0.1053	0.1024
CAAS38	2	0.4500	0.5013
CAAS39	4	0.3226	0.5960
CAAS40	3	0.0000	0.3579
CAAS41	3	0.0645	0.5812
CAAS42	5	0.7500	0.7599
CAAS43	3	0.0000	0.6400
CAAS44	4	0.3333	0.6078
CAAS45	4	0.1034	0.6068
CAAS46	3	0.0625	0.2758
CAAS47	5	0.0000	0.6885
CAAS48	3	0.5333	0.6706
CAAS49	3	0.0938	0.6424
CAAS50	4	0.2759	0.6733
CAAS51	4	1.0000	0.7270
CAAS52	3	0.7000	0.5757
CAAS53	5	0.5806	0.7832
CAAS54	5	0.6129	0.7441
CAAS55	3	0.0000	0.4504
CAAS56	2	0.5000	0.4944
CAAS57	5	0.2188	0.5045
CAAS58	3	0.4167	0.5616
CAAS59	5	0.5200	0.6686
CAAS60	3	0.8182	0.6104
CAAS61	3	0.2667	0.4881
CAAS62	2	0.6250	0.4583
CAAS63	3	0.1176	0.5704
CAAS64	4	0.4194	0.7229
CAAS65	4	0.4643	0.7266
CAAS66	4	0.3871	0.7123
CAAS67	4	0.0000	0.4719
CAAS68	2	0.2500	0.2283
CAAS69	6	0.9524	0.8072
CAAS70	2	0.0000	0.5034
CAAS71	6	0.1429	0.8097
CAAS72	2	0.1000	0.4808
CAAS73	5	0.2000	0.6220
CAAS74	3	0.1250	0.2651
CAAS75	5	0.2222	0.6797
CAAS76	4	0.1724	0.3358
CAAS77	5	0.3600	0.6106
CAAS78	5	0.6000	0.7734
CAAS79	5	0.2812	0.7941
CAAS80	4	0.6400	0.7192
CAAS81	5	0.0500	0.7167
CAAS82	4	0.6875	0.6230
CAAS83	4	0.6000	0.7590
CAAS84	3	0.0625	0.4172
CAAS85	3	0.3750	0.5928
CAAS86	3	0.0323	0.4691
CAAS87	5	0.9091	0.8139
CAAS88	6	0.8571	0.8269
CAAS89	8	0.0000	0.8410
CAAS90	4	0.5294	0.6471
CAAS91	5	0.8710	0.6267
CAAS92	4	0.3750	0.5382
CAAS93	4	0.1562	0.7217
CAAS94	5	0.2400	0.7412

Notes: Number of alleles (*Na*), expected heterozygosity (*He*) and observed heterozygosity (*Ho*).

**Figure 4:**
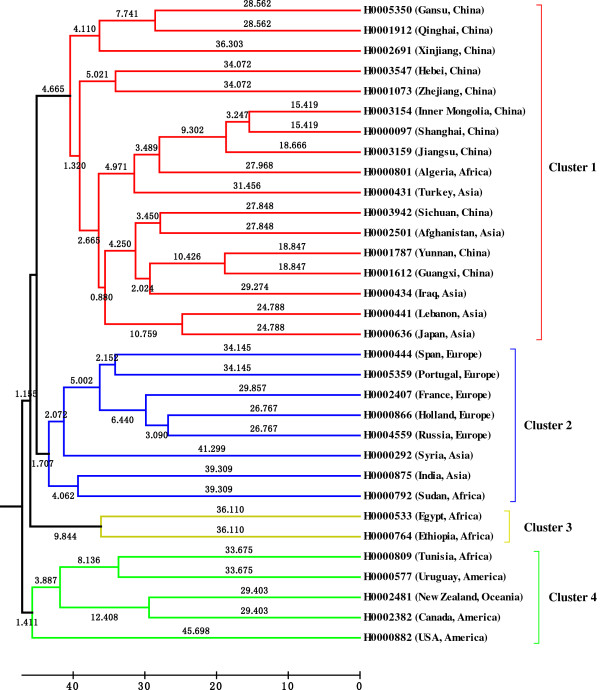
**UPGMA dendrogram of 32 genotypes of faba bean** .

The dendrogram showed that the 32 faba bean genotypes fell into four distinct clusters (Figure 4). Cluster 1 comprised accessions from China and other Asian countries except for one accessions from Africa. Cluster 2 comprised accessions from Europe and nearby regions such as Syria. Cluster 3 comprised accessions from Africa and Cluster 4 contained accessions from America, Oceania and Africa. The pattern of diversity was similar to that previously observed using AFLP [29] and ISSR [30] markers.

## Discussion

This study demonstrated that massively parallel sequencing technology offers opportunity to quickly identify large numbers of high quality SSR with diverse motifs from a genetically orphaned species such as *Vicia faba*. Given the huge number of marker loci identified in this study, future SSR marker optimisation may be best focussed on those comprising trinucleotide repeats. These repeats are generally more robust since they are reported to give fewer “stutter bands” than those based on dinucleotide repeats [31,32]. Also, trinucleotide repeats in particular have been demonstrated to be highly polymorphic and stably inherited in the human genome [33-35]. While the tri- and dinucleotide repeats mostly contributed to the major proportion of SSRs, a very small share was contributed by mono-, tetra-, penta- and hexa-nucleotide repeats. A similar trend was observed in other species [36].

The conversion of SSR-containing sequences into single locus markers may have a low success rate due to complex and/or insufficient flanking sequence. For example, just 20% of the identified dinucleotide repeats from spruce were converted to clear, discrete markers [37]. Similar observations were made for pine [38], wheat [39] and previously for *V. faba*[12]. Another factor affecting the development of clear markers is the complexity of the repeat motifs, indeed a high proportion of the SSR in the current study comprised compound repeats (49.1%). Nevertheless, this study has provided the selected data required to potentially develop tens of thousands of novel SSR markers for the faba bean genome.

Previously, a total of 304,680 reads were generated and 802 EST-SSR primer pairs were designed from transcriptome sequencing of faba bean [40]. From this, 81 primer pairs were developed, of which 48% produced polymorphic markers on the genotypes assessed. In our study, 68% (102) of the SSR loci identified were accurately amplified, of which 63% (94) were polymorphic among the genotypes tested. This may be indicatative of the larger number of SSR loci detected, inclusive of non-transcribed sequences. Hence these markers may be more representative of the entire genome for the purposes of germplasm diversity assessment and conservation purposes [41]. Meanwhile, the identification of EST-SSR within sequences provides future opportunity to mine the expressed sequences for significant physical and functional association with traits of interest in marker-assisted faba bean breeding.

## Conclusion

This work represents a major advance in the identification of large numbers of informative SSR loci in *V. faba* by application of 454 GS FLX Titanium sequencing technology.

## Abbreviations

SSR: Simple sequence repeat; QTL: Quantative Trait Locus; MAS: Marker-assisted selection; NGS: Next generation sequencing; EST: Express sequence tag; NCBI: National Center for Biotechnology Information; CTAB: Cetyltrimethylammonium bromid; MISA: Microsatellite identification; *Na*: Number of alleles; *He*: Expected heterozygosities; *Ho*: Observed heterozygosities.

## Competing interests

The authors declare that they have no competing interests.

## Authors’ contributions

TY performed bioinformatic analysis, primer design and drafted the manuscript. SYB created the SSR sequences rich DNA library, and participated in 454 sequencing. RF assisted in designing experiment and preparing the manuscript. TJJ tested SSR markers. JPG and YHH prepared all the seeds of *V. faba*. XLS and JYJ took charge of quality inspection of the DNA library. JJH and XYZ participated in conceiving the study and the manuscript drafting. XXZ designed and coordinated the study, and assisted in preparing the manuscript. All authors read and approved the final manuscript.

## Supplementary Material

Additional file 1 ** Table S1.** The information of *Vicia faba* L. germplasm used in this study. Click here for file

Additional file 2 ** Table S2. **The primer pairs were successfully designed by Primer3. Click here for file

Additional file 3 ** Figure S1.** Frequences of different SSR repeat motif types in mononuceotide. Click here for file

Additional file 4 ** Figure S2.** Frequences of different SSR repeat motif types in dinuceotide. Click here for file

Additional file 5 Frequences of different SSR repeat motif types in trinuceotide. Click here for file

Additional file 6 ** Figure S4.** Frequences of different SSR repeat motif types in tetranuceotide. Click here for file

Additional file 7 ** Figure S5. Figure S3.** Frequences of different SSR repeat motif types in pentanuceotide. Click here for file

Additional file 8 ** Figure S6. **Frequences of different SSR repeat motif types in heaxanuceotide. Click here for file

## References

[B1] Rispail N Kal P Kiss GB Ellis THN Gallardo K Thompson RD Prats E Larrainzar E Ladrera R Gonzalez EM ArreseIgor C Ferguson BJ Gresshoff PM Rubiales D Model legumes contribute to faba bean breeding Field Crop Res 2010 115 253 269 10.1016/j.fcr.2009.03.014

[B2] Duc G Marget P Esnault R Le Guen J Bastianelli D Genetic variability for feeding value of faba bean seeds (Vicia faba): Comparative chemical composition of isogenics involving zero-tannin and zero-vicine genes J Agric Sci 1999 133 185 196 10.1017/S0021859699006905

[B3] FAOSTAT 2010 http://faostat.fao.org/site/291/default.aspx

[B4] Johnston JS Bennett MD Rayburn AL Galbraith DW Price HJ Reference standards for determination of DNA content of plant nuclei Am J Bot 1999 86 609 10.2307/2656569 10330063

[B5] Morozova O Marra MA Applications of next-generation sequencing technologies in functional genomics Genomics 2008 92 255 264 10.1016/j.ygeno.2008.07.001 18703132

[B6] Mardis ER The impact of next-generation sequencing technology on genetics Trends Genet 2008 24 133 141 10.1016/j.tig.2007.12.007 18262675 PMC2680276

[B7] Lam HM Xu X Liu X Chen W Yang G Wong FL Li MW He W Qin N Wang B Li J Jian M Wang J Shao G Wang J Sun SSM Zhang G Resequencing of 31 wild and cultivated soybean genomes identifies patterns of genetic diversity and selection Nat Genet 2010 42 1053 1059 10.1038/ng.715 21076406

[B8] Huang X Wei X Sang T Zhao Q Feng Q Zhao Y Li C Zhu C Lu T Zhang Z Li M Fan D Guo Y Wang A Wang L Deng L Li W Lu Y Weng Q Liu K Huang T Zhou T Jing Y Li W Lin Z Buckler ES Qian Q Zhang QF Li J Han B Genome-wide association studies of 14 agronomic traits in rice landraces Nat Genet 2010 42 961 967 10.1038/ng.695 20972439

[B9] Tautz D Renz M Simple sequences are ubiquitous repetitive components of eukaryotic genomes Nucleic Acids Res 1984 12 4127 4138 10.1093/nar/12.10.4127 6328411 PMC318821

[B10] Lagercrantz U Ellegren H Andersson L The abundance of various polymorphic microsatellite motifs differs between plants and vertebrates Nucleic Acids Res 1993 21 1111 1115 10.1093/nar/21.5.1111 8464696 PMC309270

[B11] Rafalski JA Tingey SV Genetic diagnostics in plant breeding: RAPDs, microsatellites and machines Trends Genet 1993 9 275 280 10.1016/0168-9525(93)90013-8 8104363

[B12] Požárková D Koblížková A Román B Torres AM Lucretti S Lysák M Doležel J Macas J Development and characterization of microsatellite markers from chromosome 1-specific DNA libraries of Vicia faba Biologia Plantarum 2002 45 337 345 10.1023/A:1016253214182

[B13] Zeid M Mitchell S Link W Carter M Nawar A Fulton T Kresovich S Simple sequence repeats (SSRs) in faba bean: new loci from Orobanche-resistant cultivar ‘Giza 402’ Plant Breeding 2009 128 149 155 10.1111/j.1439-0523.2008.01584.x

[B14] Gong Y-M Xu S-C Mao W-H Hu Q-Z Zhang G-W Ding J Li Z-Y Generation and characterization of 11 novel EST derived microsatellites from Vicia faba (Fabaceae) Am J Bot 2010 97 e69 e71 10.3732/ajb.1000166 21616857

[B15] Ma Y Yang T Guan J Wang S Wang H Sun X Zong X Development and characterization of 21 EST-derived microsatellite markers in Vicia faba (fava bean) Am J Bot 2011 98 e22 e24 10.3732/ajb.1000407 21613098

[B16] Zhang WK Wang YJ Luo GZ Zhang JS He CY Wu XL Gai JY Chen SY QTL mapping of ten agronomic traits on the soybean (Glycine max L. Merr.) genetic map and their association with EST markers Theor Appl Genet 2004 108 1131 1139 10.1007/s00122-003-1527-2 15067400

[B17] Matthews BF Devine TE Weisemann JM Beard HS Lewers KS MacDonald MH Park Y-B Maiti R Lin J-J Kuo J Pedroni MJ Cregan PB Saunders JA Incorporation of sequenced cDNA and genomic markers into the soybean genetic map Crop Science 2001 41 516 521 10.2135/cropsci2001.412516x

[B18] Hisano H Sato S Isobe S Sasamoto S Wada T Matsuno A Fujishiro T Yamada M Nakayama S Nakamura Y Watanabe S Harada K Tabata S Characterization of the soybean genome using EST-derived microsatellite markers DNA Res 2007 14 271 281 18192281 10.1093/dnares/dsm025 PMC2779906

[B19] Dellaporta S Wood J Hicks J A plant DNA minipreparation: Version II Plant Molecular Biology Reporter 1983 1 19 21 10.1007/BF02712670

[B20] Doyle JJ Doyle JL A rapid total DNA preparation procedure for fresh plant tissue Focus 1990 12 13 15

[B21] Kandpal RP Kandpal G Weissman SM Construction of libraries enriched for sequence repeats and jumping clones, and hybridization selection for region-specific markers Proc Natl Acad Sci USA 1994 91 88 92 10.1073/pnas.91.1.88 8278412 PMC42891

[B22] Armour JA Neumann R Gobert S Jeffreys AJ Isolation of human simple repeat loci by hybridization selection Hum Mol Genet 1994 3 599 565 10.1093/hmg/3.4.599 8069306

[B23] Glenn TC Schable NA Isolating microsatellite DNA loci Methods Enzymol 2005 395 202 222 15865969 10.1016/S0076-6879(05)95013-1

[B24] Rice P Longden I Bleasby A EMBOSS: the European molecular biology open software suite Trends Genet 2000 16 276 277 10.1016/S0168-9525(00)02024-2 10827456

[B25] Thiel Michalek Varshney Graner Exploiting EST databases for the development and characterization of gene-derived SSR-markers in barley (Hordeum vulgare L.) Theor Appl Genet 2003 106 411 422 12589540 10.1007/s00122-002-1031-0

[B26] Yeh FC Boyle TJB Population genetic analysis of co-dominant and dominant markers and quantitative traits Belgian Journal of Botany 1997 129 157

[B27] Nei M Estimation of average heterozygosity and genetic distance from a small number of individuals Genetics 1978 89 583 590 17248844 10.1093/genetics/89.3.583 PMC1213855

[B28] Tamura K Dudley J Nei M Kumar S MEGA4: molecular evolutionary genetics analysis (MEGA) software version 4.0 Mol Biol Evol 2007 24 1596 1599 10.1093/molbev/msm092 17488738

[B29] Zong X Liu X Guan J Wang S Liu Q Paull JG Redden R Molecular variation among Chinese and global winter faba bean germplasm Theor Appl Genet 2009 118 971 978 10.1007/s00122-008-0954-5 19169661

[B30] Wang HF Zong XX Guan JJ Yang T Sun XL Ma Y Redden R Genetic diversity and relationship of global faba bean (Vicia faba L.) germplasm revealed by ISSR markers Theor Appl Genet 2012 124 789 797 10.1007/s00122-011-1750-1 22204023

[B31] Hearne CM Ghosh S Todd JA Microsatellites for linkage analysis of genetic traits Trends Genet 1992 8 288 294 1509520 10.1016/0168-9525(92)90256-4

[B32] Diwan N Cregan PB Automated sizing of fluorescent-labeled simple sequence repeat (SSR) markers to assay genetic variation in soybean Theor Appl Genet 1997 95 723 733 10.1007/s001220050618

[B33] Edwards A Civitello A Hammond HA Caskey CT DNA typing and genetic mapping with trimeric and tetrameric tandem repeats Am J Hum Genet 1991 49 746 756 1897522 PMC1683171

[B34] Gastier JM Pulido JC Sunden S Brody T Buetow KH Murray JC Weber JL Hudson TJ Sheffield VC Duyk GM Survey of trinucleotide repeats in the human genome: assessment of their utility as genetic markers Hum Mol Genet 1995 4 1829 1836 10.1093/hmg/4.10.1829 8595403

[B35] Sheffield VC Weber JL Buetow KH Murray JC Even DA Wiles K Gastier JM Pulido JC Yandava C Sunden SL Mattes G Businga T McClain A Beck J Scherpler T Gilliam J Zhong J Duyk GM A collection of tri- and tetranucleotide repeat markers used to generate high quality, high resolution human genome-wide linkage maps Hum Mol Genet 1995 4 1837 1844 10.1093/hmg/4.10.1837 8595404

[B36] Sonah H Deshmukh RK Sharma A Singh VP Gupta DK Gacche RN Rana JC Singh NK Sharma TR Genome-wide distribution and organization of microsatellites in plants: an insight into marker development in Brachypodium PLoS One 2011 6 e21298 10.1371/journal.pone.0021298 21713003 PMC3119692

[B37] Pfeiffer A Olivieri AM Morgante M Identification and characterization of microsatellites in Norway spruce (Picea abies K.) Genome 1997 40 411 419 10.1139/g97-055 9276931

[B38] Smith D Devey ME Occurrence and inheritance of microsatellites in Pinus radiata Genome 1994 37 977 983 10.1139/g94-138 7828844

[B39] Roder MS Plaschke J Konig SU Borner A Sorrells ME Tanksley SD Ganal MW Abundance, variability and chromosomal location of microsatellites in wheat Mol Gen Genet 1995 246 327 333 10.1007/BF00288605 7854317

[B40] Kaur S Pembleton LW Cogan NO Savin KW Leonforte T Paull J Materne M Forster JW Transcriptome sequencing of field pea and faba bean for discovery and validation of SSR genetic markers BMC Genomics 2012 13 104 10.1186/1471-2164-13-104 22433453 PMC3352077

[B41] Zong X Redden RJ Liu Q Wang S Guan J Liu J Xu Y Liu X Gu J Yan L Ades P Ford R Analysis of a diverse global Pisum sp. collection and comparison to a Chinese local P. sativum collection with microsatellite markers Theor Appl Genet 2009 118 193 204 10.1007/s00122-008-0887-z 18815768

