# Supplementary figures and images for: High-throughput novel microsatellite marker of faba bean via next generation sequencing
Source: BMC Genomics. 2012 Nov 8;13:602. doi: 10.1186/1471-2164-13-602 (PMC3542174; doi:10.1186/1471-2164-13-602)

Frequencies of different SSR repeat motif types in mononuceotide

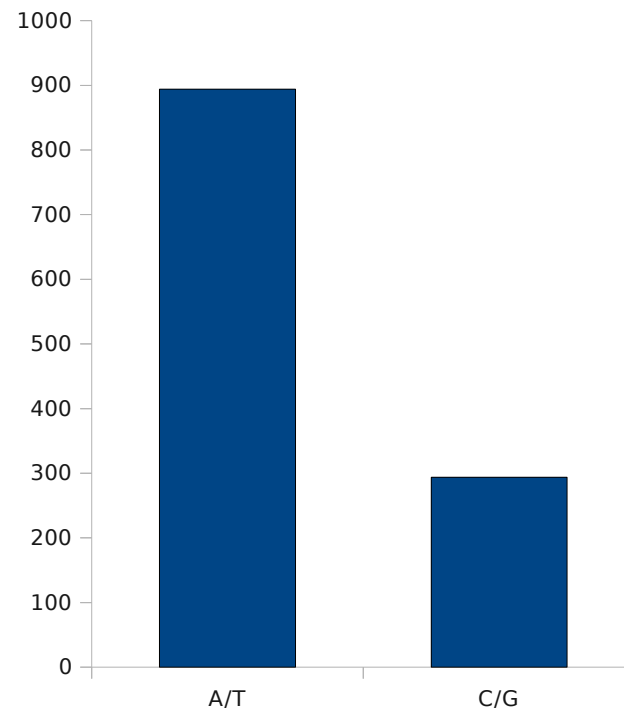

Supplement: Additional file 3 — Figure S1. Frequences of different SSR repeat motif types in mononuceotide. [file 1471-2164-13-602-S3.pdf]

Frequencies of different SSR repeat motif types in dinucleotide

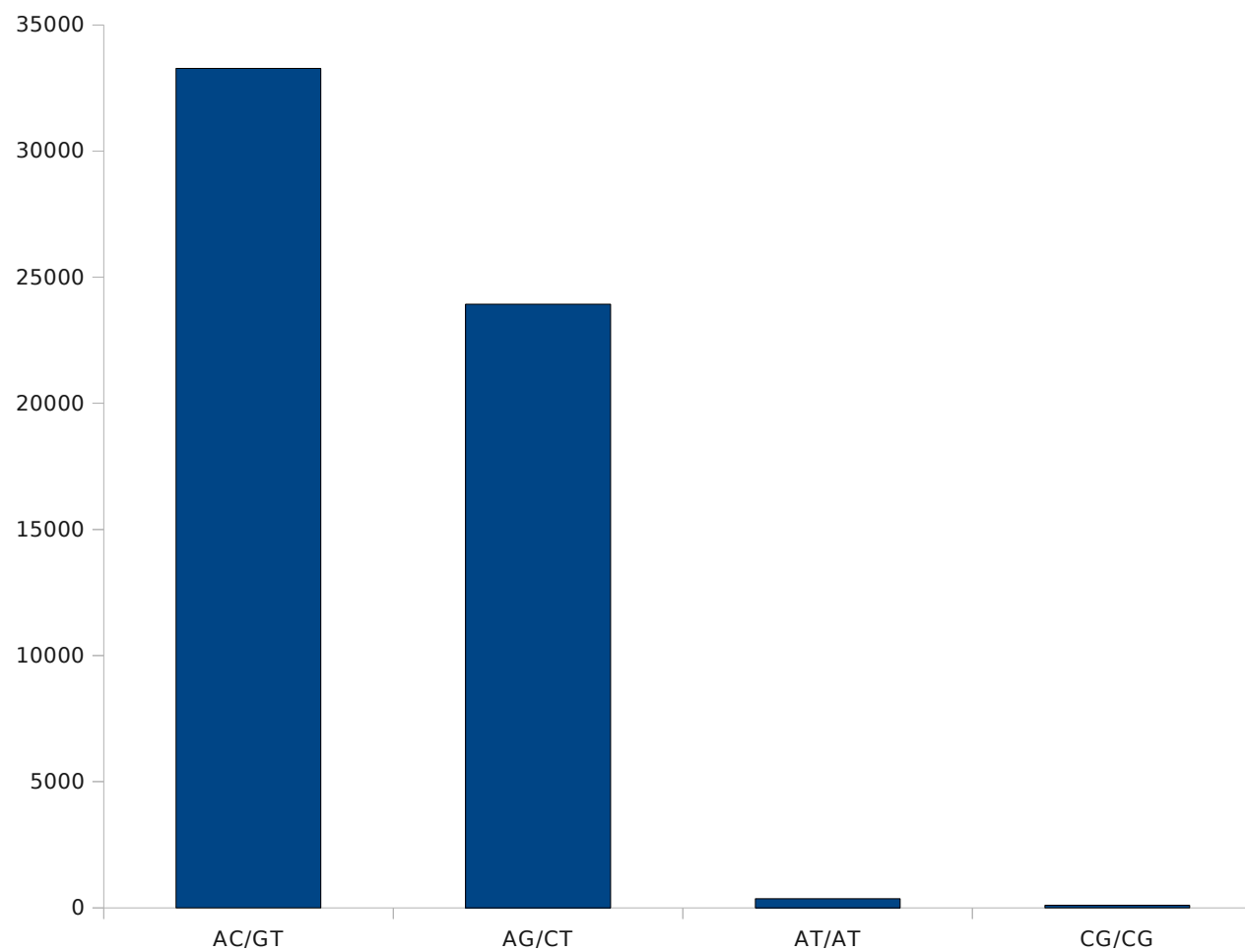

Supplement: Additional file 4 — Figure S2. Frequences of different SSR repeat motif types in dinuceotide. [file 1471-2164-13-602-S4.pdf]

Frequencies of different SSR repeat motif types in trinucleotide

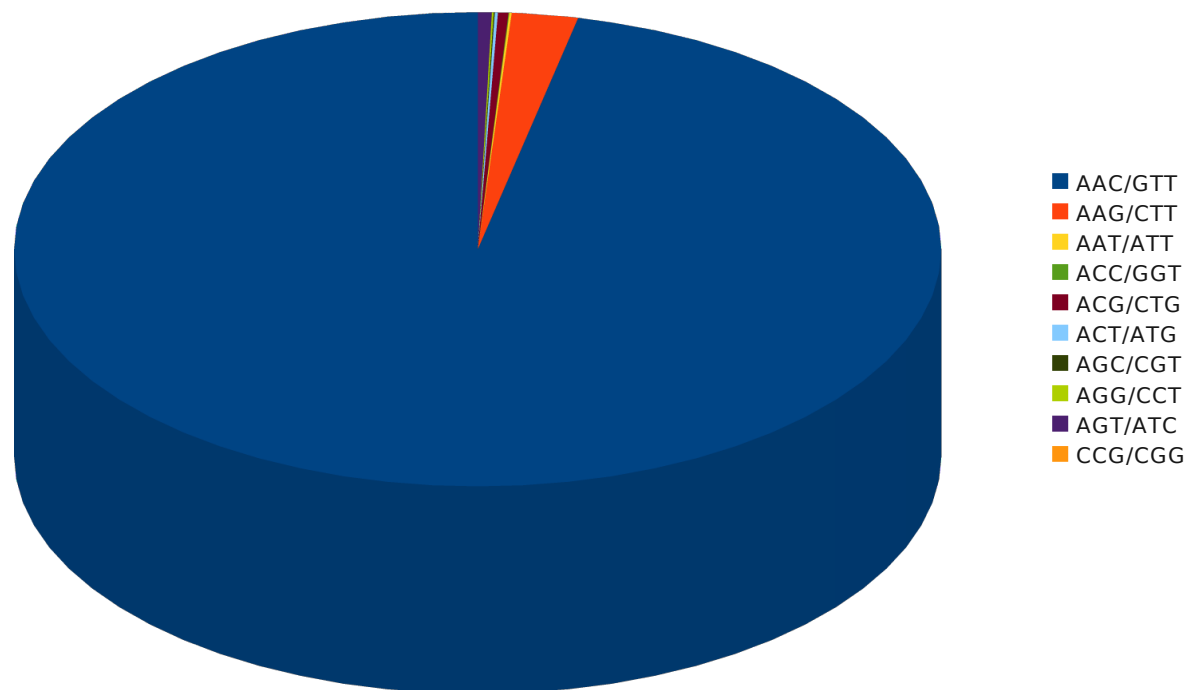

Supplement: Additional file 5 — Frequences of different SSR repeat motif types in trinuceotide. [file 1471-2164-13-602-S5.pdf]

Frequencies of different SSR repeat motif types in tetranucleotide

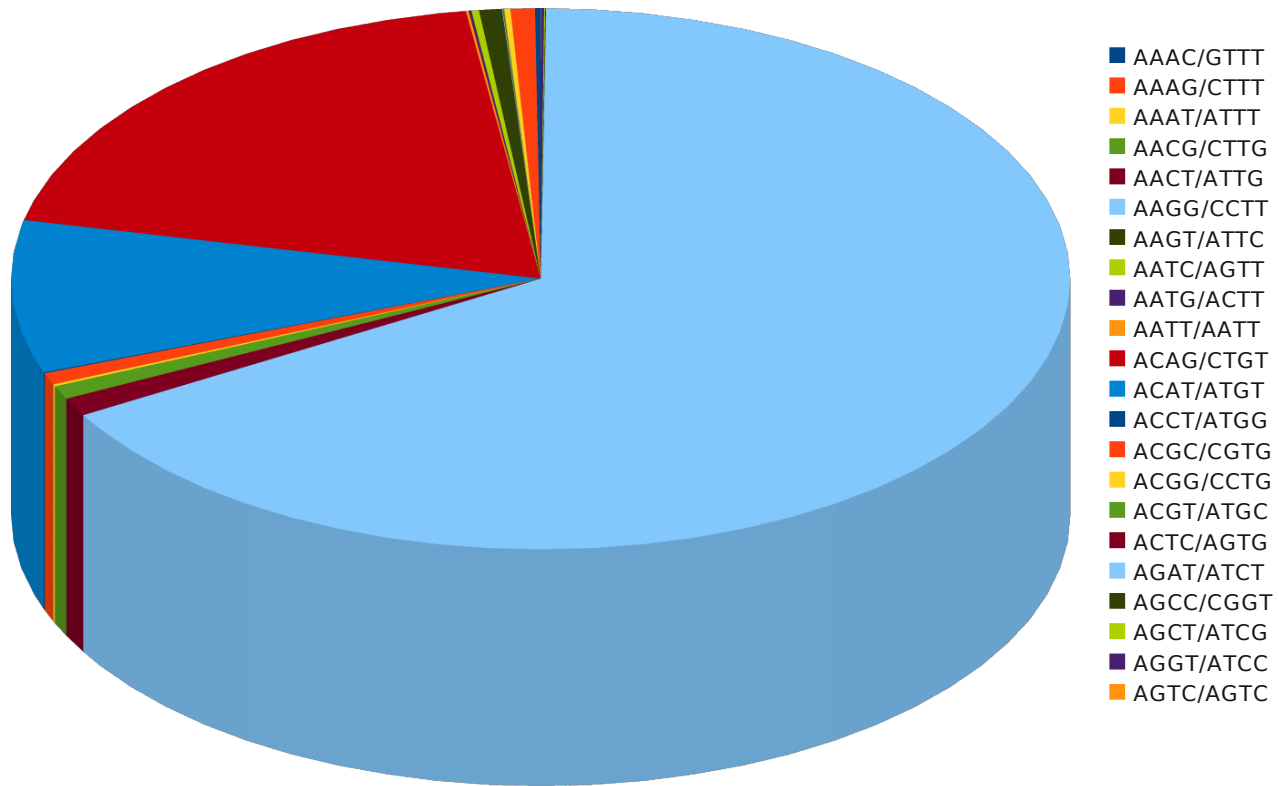

Supplement: Additional file 6 — Figure S4. Frequences of different SSR repeat motif types in tetranuceotide. [file 1471-2164-13-602-S6.pdf]

Frequencies of different SSR repeat motif types in pentanucleotide

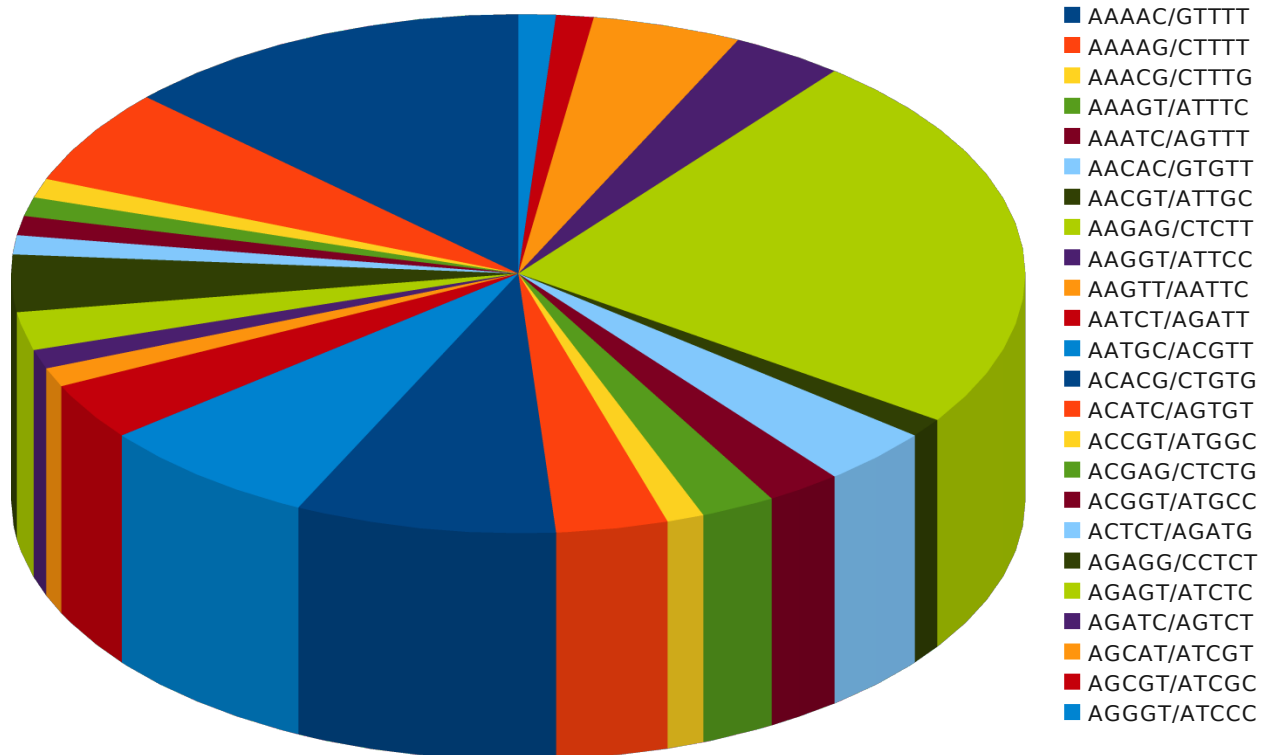

Supplement: Additional file 7 — Figure S5. Figure S3. Frequences of different SSR repeat motif types in pentanuceotide. [file 1471-2164-13-602-S7.pdf]

Frequencies of different SSR repeat motif types in heaxanucleotide

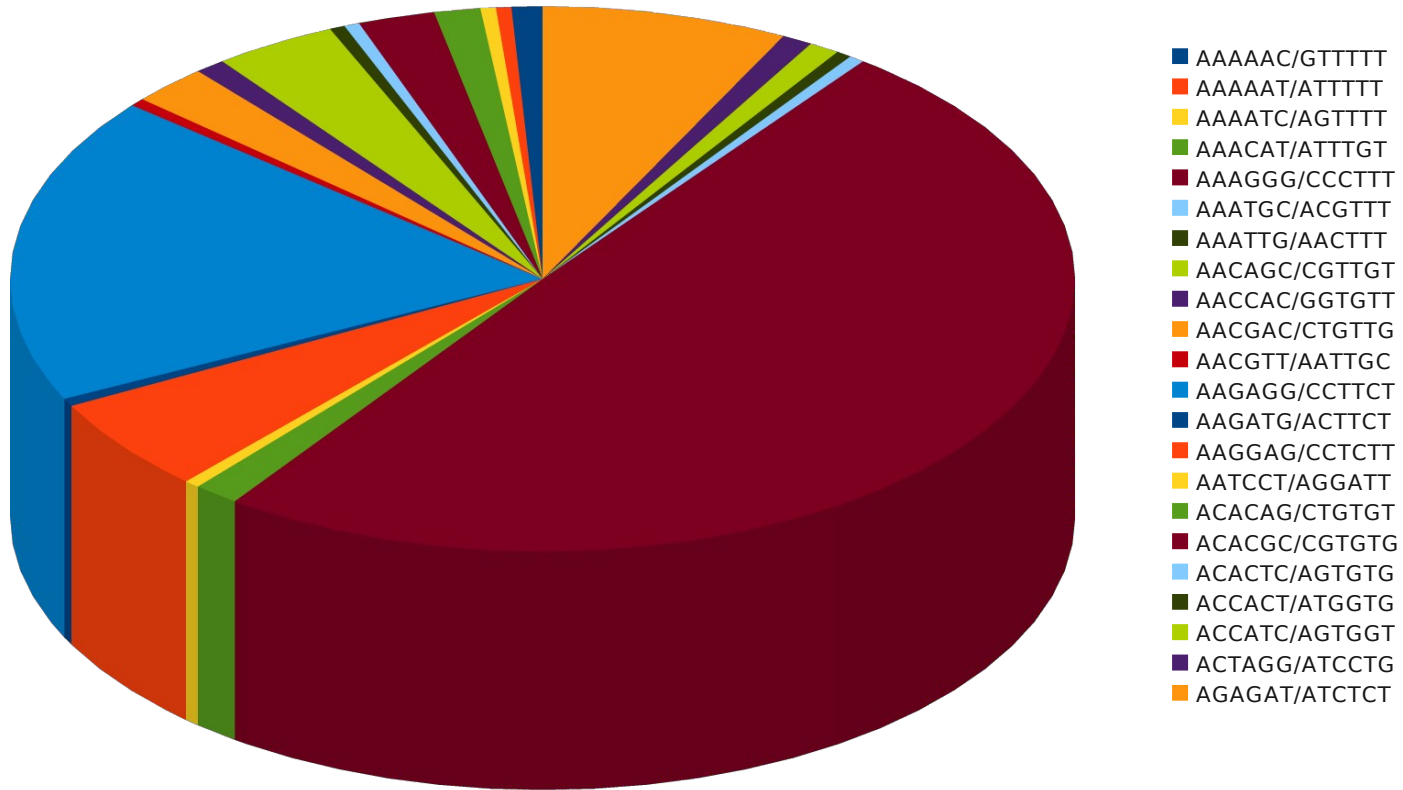

Supplement: Additional file 8 — Figure S6. Frequences of different SSR repeat motif types in heaxanuceotide. [file 1471-2164-13-602-S8.pdf]
